# Validation of a lateral flow assay for the qualitative detection of domoic acid in shellfish

**DOI:** 10.1093/jaoacint/qsag035

**Published:** 2026-04-26

**Authors:** Daud H Khan, Roozbeh Safavieh, Andrew D Turner, Ryan P Alexander, Shamina Page, Karl Dean, Rozhin Saebi, Maarij Baig

**Affiliations:** Sensoreal Inc, 6674 Esplanade Avenue (Suite B), Montreal, QC H2V 4L5, Canada; Sensoreal Inc, 6674 Esplanade Avenue (Suite B), Montreal, QC H2V 4L5, Canada; Centre for Environment, Fisheries and Aquaculture Science, The Nothe, Barrack Road, Weymouth, Dorset DT4 8UB, UK; Centre for Environment, Fisheries and Aquaculture Science, The Nothe, Barrack Road, Weymouth, Dorset DT4 8UB, UK; Centre for Environment, Fisheries and Aquaculture Science, The Nothe, Barrack Road, Weymouth, Dorset DT4 8UB, UK; Centre for Environment, Fisheries and Aquaculture Science, The Nothe, Barrack Road, Weymouth, Dorset DT4 8UB, UK; Sensoreal Inc, 6674 Esplanade Avenue (Suite B), Montreal, QC H2V 4L5, Canada; Sensoreal Inc, 6674 Esplanade Avenue (Suite B), Montreal, QC H2V 4L5, Canada

## Abstract

**Background:**

Domoic acid (DA), produced by *Pseudo-nitzschia* species during harmful algal blooms, causes amnesic shellfish poisoning (ASP) in humans. Regulatory monitoring relies on accurate but resource-intensive liquid chromatography with ultraviolet detection (LC-UV) reference method. There is urgent need for validated rapid screening tools for field and laboratory settings.

**Objective:**

This study evaluated the Sensoreal ASP Alert™ lateral flow assay (LFA) for qualitative detection of DA in bivalves through single-laboratory validation, combining AOAC and Interstate Shellfish Sanitation Conference (ISSC) guidelines.

**Methods:**

The LFA was assessed for Probability of Detection (sensitivity, specificity, false positives, false negatives), ruggedness, and comparability against LC-UV reference. Validation used spiked (mussels and scallops) and 168 naturally contaminated samples representing diverse species and origins. Additional assessments included selectivity against other marine biotoxins and semiquantitative interpretation via a SensoRead™ scanner.

**Results:**

At a 15 mg/kg decision threshold (75% of the regulatory maximum permitted limit, MPL), the LFA achieved high sensitivity (0.983) and specificity (0.817) in spiked samples, with 0.90 overall agreement (κ = 0.80). Naturally contaminated samples showed near-perfect LC-UV agreement (sensitivity 0.983; specificity 0.827; κ = 0.869). Ruggedness testing revealed no significant performance differences under varying analysts, lots, or incubation conditions. No cross-reactivity was observed with paralytic shellfish toxins (saxitoxin, gonyautoxins GTX2&3, C toxins C1&2), okadaic acid (OA), dinophysistoxin-2 (DTX2), azaspiracids-1 and -2 (AZA1&2), microcystins (MCs), and tetrodotoxin (TTX). The scanner’s Low/Medium/High classification provided early-warning capability, with substantial agreement at a secondary 7.5 mg/kg threshold (κ  =  0.667). Combined spiked and naturally contaminated data (n = 288) confirmed 0.920 overall agreement (κ  =  0.840).

**Conclusion:**

Sensoreal ASP Alert LFA is a robust, accurate, user-friendly, and rapid DA screening method. It demonstrates excellent comparability to LC-UV, high sensitivity, and rugged performance. Its semiquantitative classification aids proactive monitoring and regulatory decision-making.

**Highlights:**

A lateral flow assay for ASP has been validated successfully.

## Introduction

Domoic acid (DA) is a naturally occurring, nonprotein amino acid produced by certain species of marine diatoms of the genera *Pseudo-nitzschia* and *Nitzschia* (1). It is the principal toxin responsible for amnesic shellfish poisoning (ASP) in humans and is produced in significant quantities during the rapid proliferation of these diatoms, a phenomenon known as a harmful algal bloom (HAB) (1, 2). DA was first identified as a public health risk after a major ASP outbreak in Canada in 1987, when contaminated mussels caused gastrointestinal and severe neurological symptoms, eventually leading to disorientation, memory loss, and the death of three elderly individuals (1, 3). When these illnesses were first reported, the rapid identification of the toxin and the development of analytical methods for detecting it in shellfish were the result of an impressive, multiagency collaborative effort. This extensive response involved the National Research Council (NRC) of Canada alongside Health Canada, the Department of Fisheries and Oceans, the Centre for Disease Control, and various regional health departments. Since then, the increasing prevalence of HABs has led to frequent outbreaks worldwide, including a severe 2025 event in California that poisoned numerous marine mammal species, including sea lions and dolphins (4). Due to the significant human health risks associated with DA, regulatory authorities around the world require routine Official Control testing of live bivalve shellfish. Under most legislation (4), including European [EU regulation 627/2019 (5) and EC Regulation 853/2004 (6)], U.S. [FDA, 2020a (7)], and Canadian [Food and Drug Regulations CRC, C870, 2021 (8)], the maximum permitted limit for domoic and epi-domoic acid is 20 mg/kg, with harvesting suspended and products withheld from market (9) until levels fall below this threshold (10). These internationally recognized regulatory limits serve as a critical and effective safety standard, ensuring that consumers are well protected from the health risks associated with domoic acid ingestion.

Official control (OC) laboratories use reference methods based on liquid chromatography with ultraviolet detection (LC-UV) for regulatory testing (11). In addition to regulatory monitoring, European Union requirements stipulate that food business operators (FBOs) must perform end product testing (EPT) as part of their hazard management procedures before harvest and/or sale (12). Furthermore, a joint expert report by the Food and Agriculture Organization–Intergovernmental Oceanographic Commission (FAO-IOC) and the International Atomic Energy Agency (IAEA) recently concluded that there is an urgent need for rapid toxin test kits for industry use, which must be supported by robust scientific data describing their validation status and fitness for purpose (13). Although LC-UV is potentially suitable for EPT, it is mostly impractical for FBOs because it requires specialized laboratory facilities, costly instrumentation, and trained analytical and instrument servicing staff with expertise in quality control (QC) and quality assurance (QA). The process also typically requires overnight analysis given the time required to extract samples, prepare for analysis, and then run an instrumental sequence incorporating analytical standards, samples, and various other QC measures. This creates a clear need for simple, rapid, and portable test kits that nonspecialists can operate rapidly without expensive equipment, while still producing reliable results. Lateral flow assays (LFAs) are well suited to this role, enabling the qualitative detection of toxins directly in shellfish tissue extracts. However, due to the lack of availability of reliable commercial rapid testing tools, there is an urgent need for alternative validated methods to support rapid testing in the shellfish industry and thereby to fulfill the requirements laid down in food safety laws.

Importantly, although certain methods such as the Biosense ELISA for DA have undergone successful AOAC validation (OMA **2006.02**) and gained Interstate Shellfish Sanitation Conference (ISSC) approval, the majority of previously available LFAs and other rapid screening immunoassays were not subjected to comprehensive validation studies with publicly available performance data. Furthermore, ASP LFAs that were previously approved for “limited use” by the ISSC, such as those produced by Neogen and Jellett, are no longer commercially available, as is also the situation with the Biosense ELISA. Although other lateral flow tests currently exist on the market, such as the Aqua BC (Chile) LFA, comprehensive peer-reviewed validation data for these screening tools remain limited. This lack of transparency makes it impossible for FBOs and laboratories to determine whether a given commercial test is truly fit for purpose.

This study aims to address this gap by evaluating the Sensoreal ASP Alert™ LFA, developed in Canada by Sensoreal, for the qualitative detection of DA in bivalve mollusks. The validation followed internationally recognized guidelines from AOAC INTERNATIONAL and the ISSC (AOAC, 2014; ISSC, 2025) (14). The objective was to determine the validated performance characteristics of the Sensoreal ASP Alert LFA. In this study, the assay was evaluated for probability of detection (POD), ruggedness, and comparability. POD measures the assay’s ability to correctly identify positive samples and is determined by assessing its sensitivity (rate of false negatives) and specificity (rate of false positives) (14). Ruggedness evaluates the method’s consistency and accuracy under minor variations in analytical techniques, reagent lots, or environmental conditions encountered across different testing settings (14). Comparability refers to the degree of agreement between the results produced by the LFA assay and those obtained using the standardized LC-UV reference method (9, 14, 15). To further examine assay selectivity for DA, an interference or selectivity study was performed using nontarget toxins such as okadaic acid and saxitoxin. Collectively, these evaluations provide a comprehensive assessment of the Sensoreal ASP Alert LFA assay’s reliability and suitability for routine DA monitoring in shellfish.

## Experimental

### Principle of the method and application

The Sensoreal ASP Alert LFA assay is a rapid LFA test developed for the qualitative detection of DA in bivalve mollusks. The method operates on the principle of competitive binding. DA is first extracted from homogenized shellfish tissue using a 50% methanol solution (1:1 reagent-grade methanol:deionized water, v/v). The extract is subsequently diluted in a proprietary buffer and incubated with a lyophilized antibody–gold nanoparticle conjugate. When DA is present in the extract, it binds to the antibodies within the conjugate, thereby reducing the quantity of unbound antibody–gold complex available to interact with the test line. The mixture is then applied to the sample port of the lateral flow dipstick and migrates by capillary action along the membrane to a test line containing DA conjugated to a carrier protein. Any unbound antibody–gold conjugate binds to the immobilized DA–protein and produces a visible line. The test line intensity is inversely proportional to DA concentration, with higher toxin concentrations resulting in fainter test lines.

A control line designed to bind the conjugate independently of DA concentration appears in all valid tests, confirming proper assay function. Results are interpreted using the Sensoreal SensoRead™ scanner, which provides both a numerical test line/control line (T/C) ratio and a qualitative classification of “High,” “Medium,” or “Low” (corresponding to approximate sample concentrations of >15 mg/kg for “High,” 7.5–15 mg/kg for “Medium,” and <7.5 mg/kg for “Low”). In the validation study described here, “High” readings were considered positive, whereas “Low” and “Medium” were categorized as negative unless otherwise stated. Although the formal single-laboratory validation described herein was conducted by trained analytical personnel to establish baseline performance metrics, the assay is explicitly designed for end-users with only very basic experience, such as shellfish harvesters and food processors. Operation requires only familiarity with shellfish handling, homogenization, and simple pipetting procedures. Field deployments with food business operators have demonstrated its practical utility as a routine screening tool for detecting DA in natural samples, assisting in the closure of affected harvesting areas and ensuring contaminated products do not reach markets.

### Reagents and consumables

The Sensoreal ASP Alert LFA kit includes LFA dipstick strips, lyophilized DA–protein conjugate vials, proprietary buffer solutions, polypropylene sample tubes, and phosphate-buffered saline (PBS). The Sensoreal SensoRead scanner is required for interpreting results. Equipment and consumables not provided with the kit but necessary for operation include shellfish shucking tools, standard home-use closed-vessel blender for shellfish tissue homogenization, an analytical balance, extraction solvent (1:1 reagent-grade methanol:deionized water, v/v), an electronic timer, and adjustable laboratory pipets covering volumes of 5–20 μL, 20–200 μL, and 100–1000 μL. To expedite sample extraction, a basic benchtop centrifuge is recommended for both FBOs and laboratories; however, gravity filtration serves as a practical alternative for field applications. Additional accessories such as test-tube racks, disposable pipet tips, and a vortex mixer were also used.

For reference LC-UV analysis, the procedure of Quilliam et al., as described by Rowland-Pilgrim et al. (16, 17), was followed. All reagents were of ≥99% purity and HPLC grade. Certified reference materials (CRMs) and calibration standards were sourced from Biotoxin Metrology, National Research Council of Canada (NRCC, Halifax, Canada).

### Method protocol

Shellfish were obtained from shellfish beds around the world, including throughout Europe, New Zealand, and South America. For LFA testing, homogenized whole soft tissue from at least 10 individual shellfish per sampling site was prepared for all species. A 0.5 ± 0.01 g aliquot of homogenized tissue was weighed into a polypropylene tube, mixed with 10.0 mL of 50% methanol (1:1 reagent-grade methanol:deionized water, v/v) for 1 min, and centrifuged at 3074 for 5 min. A 10 μL aliquot of the resulting supernatant was diluted in 1 mL of proprietary buffer, and 70 μL of this diluted extract was transferred via pipet to a conjugate vial, vortex-mixed thoroughly, and incubated for 15 min at room temperature (approx. 20–23°C). The incubated mixture was applied to the absorbent pad of the test strip and allowed to develop for 15 min before being read by the SensoRead scanner. The scanner reported the qualitative classification of “High,” “Medium,” or “Low” (corresponding to approximate sample concentrations of >15 mg/kg for “High,” 7.5–15 mg/kg for “Medium,” and <7.5 mg/kg for “Low”) and the T/C ratio for each sample.

For LC-UV analysis, 2.00 ± 0.01 g of homogenized tissue was extracted with 18.0 mL of 50% methanol. Samples were vortex-mixed at 2500 rpm for 2 min and then centrifuged at 3000 g for 10 min. The resulting supernatant was decanted and filtered through a 0.2 μm glass fiber filter into amber HPLC vials. Chromatographic analysis was performed on Agilent (Manchester, United Kingdom) 1200 LC-UV systems equipped with a quaternary pump, vacuum degasser, autosampler, column oven, and diode-array detector at 242 nm. Peaks corresponding to DA and epi-DA were quantified against external calibration standards with a minimum correlation coefficient (*r*^2^) of 0.999. Results were expressed as milligrams of DA per kilogram of tissue. The limit of detection (LOD) and limit of quantitation (LOQ) of the method were 0.4 mg/kg and 1.0 mg/kg, respectively (9, 17).

### Validation study

Single-laboratory validation of the Sensoreal ASP Alert LFA followed a combined approach based on guidance from Ellison and Fearn (18), AOAC INTERNATIONAL (19), and the Interstate Shellfish Sanitation Conference (14, 20). Given the notable differences between established validation protocols, this combined approach was strategically selected to integrate the methodological rigor of the AOAC with the specific regulatory requirements of the ISSC. Ultimately, this ensures the validation data resolves current methodological uncertainties and satisfies the rigorous requirements of international regulatory bodies. Although the three approaches differ in certain respects, each includes aspects involving the evaluation of POD, ruggedness, and comparability, i.e., comparative analysis using naturally contaminated shellfish tested against the LC-UV reference method. In this study, validation was conducted using two primary matrixes, mussels and scallops, whereas the comparative LC-UV analysis was expanded to include a wider range of species such as mussels, oysters, scallops, and clams. The inclusion of these diverse primary matrixes was specifically incorporated into the study design to investigate the potential for matrix effects. Additionally, an interference or selectivity study was conducted to evaluate the ability of the method to detect DA in the presence of other marine toxins.

For classification purposes, a decision threshold of 15 mg/kg was defined, which is equivalent to 75% of the maximum permitted limit (MPL). This accounted for measurement uncertainty in both LC-UV and LFA methods, as well as variability associated with live shellfish sampling. This threshold was chosen to provide an additional safety margin for end users. Definitions of result categories used in the study are shown in Table 1.

**Table 1:** Summary of results definitions used in study.

Indicator	Definition
True positive (TP)	LC-UV ≥15 mg/kg; “High” LFA
True negative (TN)	LC-UV <15 mg/kg; “Low” or “Medium” LFA
False positive (FP)	LC-UV <15 mg/kg; “High” LFA
False negative (FN)	LC-UV ≥15 mg/kg; “Low” or “Medium” LFA
Specificity (18)	True Negatives/(True Negatives + False Positives)
Sensitivity (18)	True positives/(True Positives + False Negatives)
Observed agreement	(True Positives + True Negatives)/total number results
Cohen’s kappa (κ) (21)	(Observed agreement—expected agreement)/(1—expected agreement)

#### POD study

POD is the probability at a given concentration of obtaining a positive response by the detection method. This is not a single metric, but a combination of two parameters, sensitivity and specificity. This was evaluated using 20 DA-negative shellfish homogenates confirmed by LC-UV, representing diverse geographical origins. Spiking experiments for POD assessment were conducted with blue mussels (*Mytilus edulis*) and whole king scallops (*Pecten maximus*). Each whole tissue homogenate was subdivided into six subsamples and fortified with known concentrations of DA to achieve final tissue concentrations of 30, 20, 17.5, 10, 5, and 0 mg/kg. These concentrations were selected to span the intended analytical range of the method. All samples were processed according to the Sensoreal ASP Alert LFA protocol and analyzed with the SensoRead scanner, which reported control line intensity, T/C ratio, and qualitative classification.

The proportion of positive results was plotted against DA concentration to generate response curves, which were then used to calculate specificity, sensitivity, and agreement values as defined in Table 1. Statistical analysis for the degree of agreement was evaluated by calculating Cohen’s kappa coefficient (κ value) (21). A minimum κ value of 0.41 is required to show a moderate degree of agreement between the method and the hypothesis, with values falling in the ranges of 0.41–0.60, 0.61–0.80, 0.81–0.99, and 1.00, equating to moderate agreement, substantial agreement, near-perfect agreement, and perfect agreement, respectively.

#### Ruggedness study

Ruggedness was assessed to determine the method’s ability to withstand minor procedural or environmental variations, such as differences in analyst, reagent batch, incubation time, and ambient temperature (14, 20). These specific parameter variations were chosen to simulate realistic, minor operational deviations that operators might inadvertently introduce during routine laboratory testing. A total of 40 mussel and 40 scallop homogenates, both naturally contaminated and fortified, were analyzed twice—once under standardized conditions (Batch A) and once under altered conditions (Batch B), as summarized in Table 2.

**Table 2:** Summary of experimental parameters used during ruggedness testing of Sensoreal DA LFA in mussels and scallops.

Parameter	Batch A	Batch B
Conjugate lot	2460773_40DAAB	2460774_40DAAB
Ambient temperature	23°C	20°C
Analyst	Analyst 1	Analyst 2
Diluent solvent batch	1	2
Strip incubation time	15 min	18 min
Day	Day 1	Day 7

Results were then compared to determine whether the minor changes made were affecting the method or not. The number of agreements between the results generated by the two assessment batches were calculated and a two-sided statistical test comparing the proportions of positive and negative results produced by the two batches was undertaken to determine significance at the 0.05 level, through calculation of the “observed contrast pair” using the *z*-test for proportions. Specifically, the proportions of positive results from each batch and the pooled proportion were used to calculate pooled proportion *p* and subsequently the *z*-test for proportions following:


$$p=\frac{p_{1}-{\;p}_{2}\;}{n_{1}-{\;n}_{2}}$$

and


$$z=\frac{p_{1}-{\;p}_{2}}{\sqrt{p(1-p)(\frac{1}{n_{1}}+\frac{1}{n_{2}})}}$$

where *p* = pooled proportion, *p*_1_ and *p*_2_ are the positive proportions of the two batches, and *n*_1_ and *n*_2_ are the sample sizes of the two batches.

The *z*-value for proportions was then compared against the *P*-value from a standard normal distribution table (two-tailed, 95% confidence) to determine significance of any difference between the two batches.

#### Comparability with LC-UV reference method

A total of 168 naturally contaminated shellfish samples were obtained from Europe, North America, New Zealand, and South America, representing a wide range of species including mussels (*M. edulis, M. galloprovincialis, M. chilensis, M. edulis platens*is, *Perna canaliculus*), scallops (*Pecten maximus, Aequipecten opercularis, Placopecten magellanicus*), cockles (*Cerastoderma edule*), clams (*Mercenaria mercenaria, Ruditapes philippinarum, Ensis* sp., *spisula solida*), and oysters (*Magallana gigas, Ostrea edulis*). DA concentrations ranged from 1 mg/kg to 353 mg/kg, exceeding 17 times the MPL.

Qualitative LFA classifications (“High,” “Medium,” “Low”) were compared against LC-UV quantifications to determine the number of true positives, true negatives, false positives, and false negatives. These data were then used to calculate sensitivity and specificity for the naturally contaminated sample set. Additionally, scanner-reported T/C ratios were plotted against LC-UV concentrations to evaluate the correlation between the two methods and to investigate the potential for semiquantitative estimation of DA concentration from LFA results.

#### Selectivity testing with interfering toxins

Selectivity was examined to assess the potential for cross-reactivity with nontarget toxins. Fourteen DA-positive samples and 14 samples containing nontarget toxins were prepared, each tested in both mussel and scallop matrixes. Nontarget analytes included paralytic shellfish toxins (saxitoxin, gonyautoxins GTX2&3, and C toxins C1&2), okadaic acid (OA), dinophysistoxin-2 (DTX2), azaspiracids-1 and -2 (AZA1&2), microcystins (MCs), and tetrodotoxin (TTX). Duplicate analyses were performed, and results from the Sensoreal ASP Alert LFA were used to determine the method’s response to target versus nontarget compounds.

## Results

### POD analysis

Raw data generated from the specificity study are tabulated in Appendix 1. The dose-response curve (Figure 1) shows a strong correlation between DA concentrations in shellfish tissue and the percentage of LFA positive results; 100% of all LFA results were positive at the 20 mg/kg DA concentration level and negative at the 0 mg/kg and 5 mg/kg levels.

**Figure 1:**
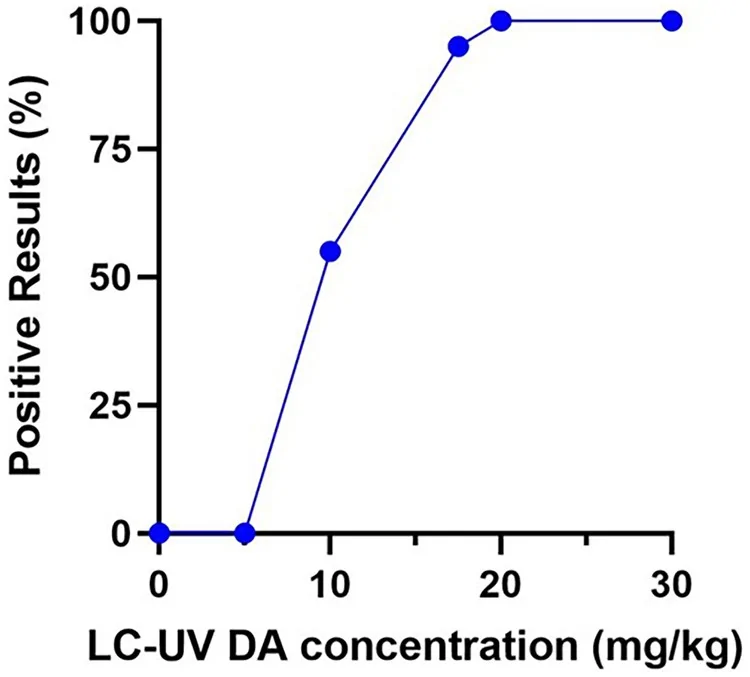
Dose–response curve for the Sensoreal ASP Alert LFA, illustrating the concentration-dependent increase in positive results for DA.

From all tests conducted using spiked mussel and scallop samples at the six concentrations at the selected threshold of 15 mg/kg DA, performance values were determined. Sensitivity was 0.983, corresponding to ∼9% false positives, specificity was 0.817, corresponding to ∼2% false negatives, which resulted in an overall agreement of 0.90 and a Cohen’s kappa (κ) of 0.80.

Table 3 provides a summary of the results obtained from the LFA analysis of both mussel and scallop matrixes, whereas Table 4 provides a detailed breakdown of the results. Taking the positive/negative threshold of 15 mg/kg, the results show an excellent level of agreement between the expected spiked concentration and the LFA result. Sensitivity values of 1.000 and 0.967 for mussels and scallops, respectively, evidences a very low proportion of false negatives, with only one scallop sample returning a “Medium” result for a sample spiked at 17.5 mg/kg. The combined sensitivity of 0.983 shows a low overall false positive rate, with the majority of these in mussels. The combined matrix agreement of 0.900 was found to equate to a kappa K-statistic of 0.800 for both matrixes, which evidences a substantial agreement between the two methods (21).

**Table 3:** Summary of POD study data generated following spikes of mussel and scallop matrixes, with results calculated based upon the 15 mg/kg threshold.

Parameter	Mussels	Scallops	Combined
Total analyses	60	60	120
True positives (TP)	30 (50%)	29 (48%)	59 (98%)
True negatives (TN)	21 (35%)	28 (47%)	49 (82%)
False positives (FP)	9 (15%)	2 (3%)	11 (9%)
False negatives (FN)	0 (0%)	1 (1.7%)	1 (2%)
Sensitivity	1.000	0.967	0.983
Specificity	0.700	0.933	0.817
Agreement [P_0_]	0.850	0.950	0.900
Kappa [κ]	0.700	0.900	0.800

**Table 4:** Detailed breakdown of the results obtained from POD study showing shellfish species, DA concentrations (mg/kg), number of tests, numbers of positive and negative samples, and true/false results.

Species	DA Concentration	N	Pos.	Neg.	TP	TN	FP	FN
Mussels	30	10	10	0	10	0	0	0
	20	10	10	0	10	0	0	0
	17.5	10	10	0	10	0	0	0
	10	10	9	1	0	1	9	0
	5	10	0	10	0	10	0	0
	0	10	0	10	0	10	0	0
Scallops	30	10	10	0	10	0	0	0
	20	10	10	0	10	0	0	0
	17.5	10	9	1	9	0	0	1
	10	10	2	8	0	8	2	0
	5	10	0	10	0	10	0	0
	0	10	0	10	0	10	0	0

N = number of replicates; Pos. = number of positive results; Neg. = number of negative results; TP = true positive; TN = true negative; FP = false positive; FN = false negative.

### Ruggedness

Raw data from the ruggedness assessment conducted using 40 samples in two batches are presented in the Appendix. Samples collected were divided into four groups based on the concentration levels of DA, specifically 0–9 mg/kg, 10–14 mg/kg, 15–19 mg/kg, and 20–30 mg/kg. The results obtained from both analytical batches conducted 7 days apart using different analysts and under different conditions were highly consistent, showing that the method parameter variations introduced in Batch B had minimal impact on the outcome. Supplementary Table S1 provides a detailed breakdown of the results obtained, whereas Table 5 provides a summary of the data, showing comparable proportions of qualitative results between the two batches of duplicate sample assessments. The *z*-test for proportions conducted on the data obtained from mussel samples yielded a *z*-value of −0.69, corresponding to a *P*-value of 0.613, based on the standard normal distribution table, confirming there were no significant differences between the results of the two batches. For scallops, the *z*-test result was 0, corresponding to a *P*-value of 0.425 also confirming no statistical difference between batches. With results from both species combined, the *z*-test *P*-value of 0.49 evidenced no statistical difference in the performance of the LFA when run under different conditions.

**Table 5:** Summary of ruggedness results obtained for mussels and scallops.

Species	Mussels		Scallops	
Batches	Batch A	Batch B	Batch A	Batch B
Number of positive results	23	26	17	17
Number of negative results	17	14	23	23
Proportion of positive results	0.58	0.65	0.43	0.43
Pooled proportion, *p*	0.613		0.425	
*z*-test for proportions, *z*	−0.69		0.0	
*P*-value (two-tailed, 95% confidence)	0.490			

### Comparability with LC-UV reference method

The data obtained from the comparability study conducted on natural samples demonstrated a strong correlation between the LFA results and the DA concentrations determined by the LC-UV reference method. To illustrate the method’s empirical probability of detection with naturally contaminated samples, it was observed that all 103 mussel and scallop samples containing higher than 18.9 mg/kg DA gave 100% positive results by the Sensoreal ASP Alert LFA. All 22 samples containing less than 8 mg/kg DA gave 100% negative results. The remaining 43 scallops, mussels, clams, and oysters containing 8 to 19 mg/kg gave a mix of positive and negative LFA results. Out of the 33 samples containing <10 mg/kg, 30 of these (91%) returned negative LFA results, either “Low” or “Medium” designations. From the 19 samples containing DA between 10 and 15 mg/kg DA, six returned positive LFAs, with the remaining 13 showing negative, comprising 85% designated “Medium” LFA results.

#### Positive/negative (high) 15 mg/kg threshold assessment

Figure 2A shows the distribution of the samples collected, whereas Figure 2B depicts their distribution according to the T/C ratio values obtained. The various samples collected were broadly classified into five shellfish families (Figure 2A), including mussels (both blue mussels and Mediterranean mussels), cockles, clams (hard clams, surf clams, Manila clams, and razors), scallops (king, Atlantic, and queen scallops), and oysters (Pacific oysters and native oysters). The ASP LFA demonstrated a high degree of accuracy in detecting varying DA levels across all shellfish species tested (Figure 2B). This performance is further supported by the close agreement between the overall distribution of natural samples and the dose–response curve generated using dilutions prepared from a commercial certified calibration solution (pure domoic acid in solvent), NRC-CRM-DA, with corresponding *R*^2^ values of 0.84 for natural samples and 0.98 for DA calibration solution. Table 6 provides a detailed breakdown of the results.

**Figure 2:**
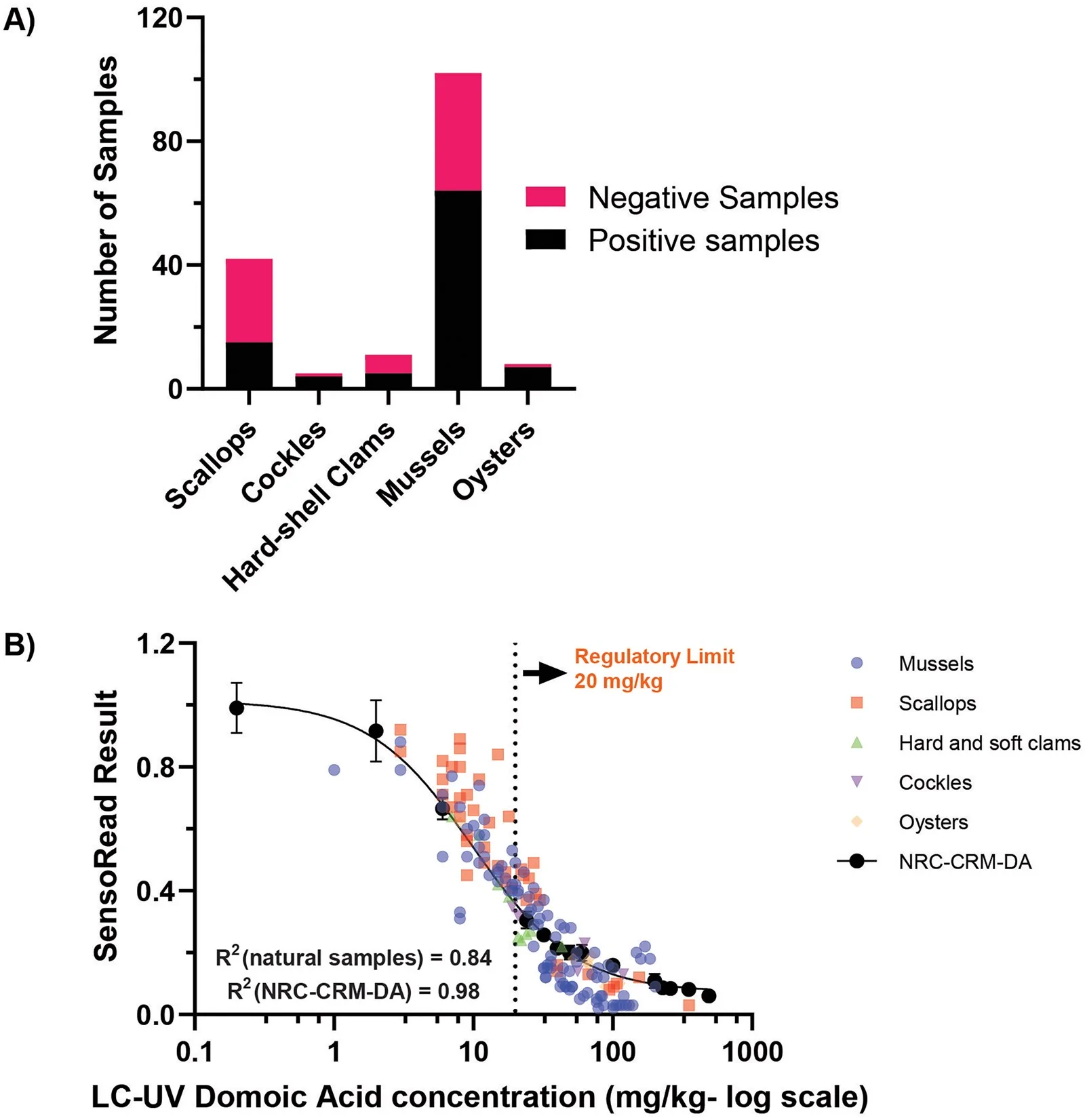
(A) Distribution of the naturally contaminated samples collected for comparability testing; (B) Sample distribution with respect to scanner result.

**Table 6:** Detailed breakdown of naturally contaminated samples and the distribution of the results obtained from Sensoreal ASP Alert LFA.

Shellfish group	Specific shellfish	Quantity	TP	TN	FP	FN
Scallops	Atlantic scallops	2	0	2	0	0
	King scallops	36	17	16	2	1
	Queen scallops	4	3	1	0	0
Cockles	Cockles	5	5	0	0	0
Hard clams	Quahog	2	2	0	0	0
	Manila clams	1	1	0	0	0
	Razor clams	7	2	4	1	0
	Surf clams	1	1	0	0	0
Mussels	Blue mussels	102	77	18	6	1
Oysters	Native oysters	2	2	0	0	0
	Pacific oysters	6	5	1	0	0

Based on the 15 mg/kg threshold, a total of 168 samples were evaluated in the comparative study. The assay correctly identified 114 true positive and 43 true negative samples. The sensitivity of the LFA from this study was 0.983, showing an excellent ability of the method to identify positive samples, given the extremely low number of false negative results (2) in relation to the 15 mg/kg threshold. However, as shown in the raw data (*see*  Appendix), both these samples deemed to be false negatives in comparison with the 15 mg/kg threshold still contained DA concentrations lower than the actual MPL of 20 mg/kg (17.9 and 18.9 mg/kg). As such, in relation to the legal MPL, the LFA produced zero false-negative results across the sample set. The specificity of the LFA was 0.827, showing a good ability of the test to identify negative samples, with just nine false positive results determined in relation to the 15 mg/kg limit applied in this study. These data showed a total agreement [P_0_] of 0.935 and a kappa value [κ] of 0.869, which equates to a near-perfect agreement between methods (21).

#### Medium 7.5 mg/kg threshold assessment

While the primary decision threshold is the 15 mg/kg positive/negative limit, a secondary “Low”/“Medium” threshold was also introduced to provide greater resolution around sub-threshold DA concentrations. This cutoff was set at a value corresponding to 7.5 mg/kg, or half the primary limit. A comparative analysis of 66 naturally contaminated samples containing 0–20 mg/kg DA (as determined by LC-UV) yielded 43 true secondary positives (TSPs) and 12 true secondary negatives (TSNs), alongside six false secondary positives (FSPs) and five false secondary negatives (FSNs). Consequently, the assay demonstrated high sensitivity (0.896) at the lower secondary threshold, with a strong probability that samples at or above 7.5 mg/kg would yield a “Medium” designation. Likewise, specificity was acceptable (0.667), with a fairly high probability of “Low” results for samples below this limit. The overall agreement [*P*_0_] between methods at the secondary threshold was 0.833, while the kappa [κ] statistic of 0.667 confirmed a “substantial agreement” in performance (21). These findings highlight the robustness of Sensoreal ASP Alert LFA in detecting and categorizing DA levels near to and below the regulatory action limit, providing additional confidence in its application as a useful screening tool.

#### Overall agreement

The data obtained from both the spiked specificity samples and the comparative testing of naturally contaminated shellfish were combined into a single dataset to evaluate the overall performance of the Sensoreal ASP Alert LFA against the reference LC-UV method. As summarized in Table 7, the combined dataset of 288 samples demonstrated a sensitivity of 0.983, specificity of 0.821, and overall agreement [P_0_] of 0.920 and a kappa [κ] agreement of 0.840. Data therefore indicated a near-perfect agreement between the two methods. Importantly, this strong performance across the different shellfish species confirmed that the diverse tissue compositions did not have a negative impact on the LFA’s overall reliability or accuracy.

**Table 7:** Summary of Sensoreal ASP Alert LFA performance based on all spiked and naturally contaminated sample analysis.

Parameter	Result
Total number of samples	288
True positives (TP)	173
True negatives (TN)	92
False positives (FP)	20
False negatives (FN)^a^	3
Sensitivity	0.983
Specificity	0.821
Agreement [P_0_]	0.920
Kappa [κ]	0.840

a Based on 15 mg/kg threshold. 0% FN determined in relation to regulatory limit of 20 mg/kg.

## Discussion

The single-laboratory validation reported in this study confirmed that the Sensoreal ASP Alert LFA is a reliable and effective method for the qualitative detection of DA in bivalve mollusk shellfish. At the regulatory action limit of 20 mg/kg, all samples tested positive. When applying a more conservative target action limit of 15 mg/kg to account for potential measurement uncertainty, including the variability inherent in shellfish sampling, only one sample returned a negative (“Medium”) result at 17.5 mg/kg. No false positives were recorded in either unspiked samples or those spiked at 5 mg/kg. Based on spiked samples, the overall agreement with the reference LC-UV method was strong. The high sensitivity (0.983) highlights the method’s excellent ability to correctly identify positive samples, ensuring that potentially contaminated shellfish are detected with minimal oversight. The specificity value (0.817), although slightly lower than sensitivity, indicates a robust ability to correctly identify negative samples, thereby minimizing unnecessary rejections of safe shellfish batches, which would otherwise result in unwarranted economic losses for producers. The overall agreement of 0.90 confirmed consistency of the method when compared with the reference LC-UV method as applied to spiked samples. The κ value of 0.80 indicates substantial agreement, according to Landis and Koch’s benchmark scale, further supporting the reliability of the method for regulatory and field applications.

Validation experiments conducted to assess the qualitative ruggedness of the LFA further showed that performance was consistent across different test kit lots, reagent batches, and conjugate batches and over different days when using different analysts. Specifically, no statistically significant differences were observed when LFA cassette incubation time was extended by +3 min beyond the standard 15-min protocol, nor between different operators or when testing was repeated one week later. Extensive testing of multiple species of 168 naturally contaminated mussel, scallop, oyster, and clams also demonstrated excellent agreement with the LC-UV reference method. Using the 15 mg/kg threshold, the calculated agreement (P_0_) was 0.935 and kappa statistic was 0.869, indicating near-perfect agreement between methods. During the evaluation, three false-negative results were observed relative to the assay’s 15 mg/kg threshold. One originated from the spiked dataset at 17.5 mg/kg, whereas the remaining two were from the naturally contaminated dataset at 17.9 and 18.9 mg/kg. Notably, because all three concentrations remained below the legal maximum permissible limit of 20 mg/kg, the assay yielded zero false-negative outcomes from a regulatory perspective.

In addition to the primary positive/negative threshold, the SensoRead scanner offers the added benefit of distinguishing between “Low,” “Medium,” and “High” results. At the secondary “Low”/“Medium” threshold of 7.5 mg/kg, the LFA achieved a sensitivity of 0.896, specificity of 0.667, and overall agreement of 0.833, with a kappa statistic of 0.667, demonstrating substantial agreement with the reference method. This capability provides valuable context for end-users, because “Medium” results may serve as an early warning of increasing DA levels during toxic phytoplankton blooms, prompting more frequent monitoring; conversely, “Low” results indicate little to no DA presence, requiring no additional action by end-users beyond continued routine testing.

When the data from both spiked and naturally contaminated samples were combined into a single dataset, the overall agreement across all 288 samples was 0.920, with a kappa statistic of 0.840, evidencing near-perfect alignment with the LC-UV method. These findings confirm that the Sensoreal ASP Alert LFA is accurate, robust, and widely applicable to multiple shellfish species, including mussels, scallops, oysters, cockles, and clams. The method is designed for use in both laboratory and field environments. It can be performed without extensive laboratory equipment and with minimal training, delivering results within 20 min of sample extraction. The assay provides a qualitative classification of DA concentrations into “High,” “Medium,” and “Low” categories. Compared to a simple positive/negative test, this classification offers a more nuanced assessment of toxin trends to support shellfish monitoring and management decisions.

## Conclusion

The single-laboratory validation study reported here demonstrated that the Sensoreal ASP Alert LFA is a robust, accurate, and user-friendly tool for the detection of DA in bivalve mollusks. The assay achieved near-perfect agreement with the LC-UV reference method, showing excellent sensitivity, good specificity, and no cross-reactivity with other common marine biotoxins. Results were consistent across different reagent lots, operators, and incubation variations, highlighting the method’s ruggedness and reliability. The ability of the LFA reader to provide “High,” “Medium,” and “Low” classifications, in addition to positive/negative determinations, offers users valuable semiquantitative insight into toxin levels. This unique feature enhances its utility as a screening tool, enabling shellfish farmers, regulators, and laboratories to detect contamination early, adjust monitoring frequency, and support timely decision-making during harmful algal bloom events. Taken together, the data support the suitability of the Sensoreal ASP Alert LFA as an effective and practical method for routine shellfish safety monitoring in both laboratory and field settings.

## Supplementary Material

qsag035_Supplementary_Data
