# Supplementary material for: Validation of a lateral flow assay for the qualitative detection of domoic acid in shellfish
Source: J AOAC Int. 2026 Apr 26;109(6):951–8. doi: 10.1093/jaoacint/qsag035 (PMC13732694; doi:10.1093/jaoacint/qsag035)
Supplement: Supplementary file 1 [file qsag035_supplementary_data.docx]

**Supplementary**

***Table S1:*** *Comparison of LFA performance results for ruggedness batches A (standard) and B (altered) across shellfish species and domoic acid concentration ranges.*

| **Species** | **DA concentration** | **N** | **Batch A** | | | | **Batch B** | | | |
| --- | --- | --- | --- | --- | --- | --- | --- | --- | --- | --- |
|  |  |  | **Pos** | **Neg** | **FP** | **FN** | **Pos** | **Neg** | **FP** | **FN** |
| Mussels | 0-9 | 12 | 0 | 12 | 0 | 0 | 0 | 12 | 0 | 0 |
|  | 10-14 | 4 | 0 | 4 | 0 | 0 | 2 | 2 | 2 | 0 |
|  | 15-19 | 11 | 10 | 1 | 0 | 1 | 11 | 0 | 0 | 0 |
|  | 20-30 | 13 | 13 | 0 | 0 | 0 | 13 | 0 | 0 | 0 |
| Scallops | 0-9 | 15 | 0 | 15 | 0 | 0 | 0 | 15 | 0 | 0 |
|  | 10-14 | 7 | 2 | 5 | 2 | 0 | 1 | 6 | 1 | 0 |
|  | 15-19 | 7 | 4 | 3 | 0 | 3 | 5 | 2 | 0 | 2 |
|  | 20-30 | 11 | 11 | 0 | 0 | 0 | 11 | 0 | 0 | 0 |

N = number of tests; Pos = number of positive results; Neg = number of negative results; TP = True Positive; TN = True Negative; FP = False Positive; FN – False Negative
